# Novel silicon bipodal cylinders with controlled resonances and their use as beam steering metasurfaces

**DOI:** 10.1038/s41598-021-93041-x

**Published:** 2021-07-01

**Authors:** Samar M. Fawzy, Ahmed M. Mahmoud, Yehea I. Ismail, Nageh K. Allam

**Affiliations:** 1 Department of Electronics and Communications Engineering, School of Sciences and Engineering, The American University in Cairo, Cairo, 11835 Egypt; 2 Center of Nanoelectronics and Devices (CND), Zewail City of Science Technology and Innovation, Cairo, 12578 Egypt; 3 Energy Materials Laboratory, School of Sciences and Engineering, The American University in Cairo, Cairo, 11835 Egypt

**Keywords:** Materials science, Nanoscience and technology, Optics and photonics, Physics

## Abstract

Metasurfaces have paved the way for high performance wavefront shaping and beam steering applications. Phase-gradient metasurfaces (PGM) are of high importance owing to the powerful and relatively systematic tool they offer for manipulating electromagnetic wave fronts and achieving various functionalities. Herein, we numerically present a novel unit cell known as bipodal cylinders (BPC), made of Silicon (Si) and placed on a Silicon dioxide (SiO_2_) substrate to be compatible with CMOS fabrication techniques and to avoid field leakage into a high index substrate. Owing to its geometrical structure, the BPC structure provides a promising unit cell for electromagnetic wave manipulation. We show that BPC offers a way to shift the electric dipole mode to a frequency higher than that of the magnetic dipole mode. We investigate the effect of varying different geometrical parameters on the performance of such unit cell. Building on that, a metasurface is then presented that can achieve efficient electromagnetic beam steering with high transmission of 0.84 and steering angle of 15.2°; with very good agreement with the theoretically predicted angle covering the whole phase range from 0 to 2$\pi$.

## Introduction

Throughout the last two decades, metamaterials have attracted extensive research efforts. Typically, metamaterials started out as three dimensional (3D) periodic subwavelength metallic/dielectric structures that resonantly couple to the electromagnetic waves, leading to unprecedented or unusual properties and features that cannot be achieved using conventional materials. Due to the usually associated high losses and strong dispersion and spectral dependence associated with the resonant responses, in addition to the difficulty of fabricating 3D metamaterials, practical applications were relatively limited. On the other hand, metasurface is termed as the category of metamaterials that inherent all the properties of metamaterials while providing a solution to the limitations of such structures. Metasurfaces are subwavelength two dimensional (2D) or quasi 2D structures that provide means to manipulate the amplitude, phase, or polarization of a propagating electromagnetic wave^1^. Phase-gradient metasurfaces exploit phase accumulation along the transverse directions to manipulate the incident wave front.  In addition, dielectric metasurfaces provide a more efficient option compared to their plasmonic metallic counterparts, owing to their low Ohmic losses, and minimal back scattering^2^. Most regular shaped dielectric metasurfaces such as spheres, cubes, cylinders, and rods use the lowest order Mie-type resonances namely magnetic and electric dipoles (MD and ED respectively) to engineer the desired phase gradient^3,4^. Moreover, the usually high refractive index can lead to strongly confined fields with minimal dissipation. Thus, they offer a great domain for numerous applications including but not limited to beam steering/focusing^5–7^, vortex generation^8,9^, holograms^5,10^, and sensing^11^. The geometrical structure of the utilized unit cell is a key factor in designing a metasurface. For example, cylinders have shown to be very efficient light scatterers^12^. It has also been demonstrated that by engineering the structure^13^, periodicity^14^, dimensions^15^, and materials^16^ of cylinders it is possible to achieve and control the position of the resonant modes. In this work, we present a novel perturbed cylindrical structure known as BPC. The complex geometry of BPCs provides several degrees of structural freedom, which can be manipulated to be used in different applications. We propose this structure as the building unit cell for a high-performance beam steering PGM.

## Methods

The BPC is drawn using Autodesk Inventor software and has height (H) of 0.7 , the outer diameter of the bottom legs as well as the top one (Dout) are 0.8 microns (µm) each, with inner diameter (Din) of 0.2 µm . For the sake of comparison, we also consider a cylinder with a hole of height (h) of 0.7 µm, outer and inner diameters (Dout and Din) of 0.8 µm and 0.2 µm, respectively. The curved part is drawn using a radius of curvature (RC) of 0.5, the bottom legs are of height (h_b) 0.1 µm are separated by distance (L) of 0.45 µm^17^. Both structures are made from Silicon (refractive index 3.67). The substrate in both cases is a cube of SiO_2_ (refractive index = 1.45) whose side length (S) is 1.7 µm. In this study, the frequency domain solver of CST Microwave Studio was used to spectrally characterize both structures’ performances when comprising a unit cell of an infinitely extended metasurface. This was achieved using Floquet ports, and unit cell boundary conditions in the x and y directions. The main mode of concern is TE(0,0) unless otherwise stated, the E field is along the y-axis.

## Results and discussion

### BPC resonances

**Figure 1:**
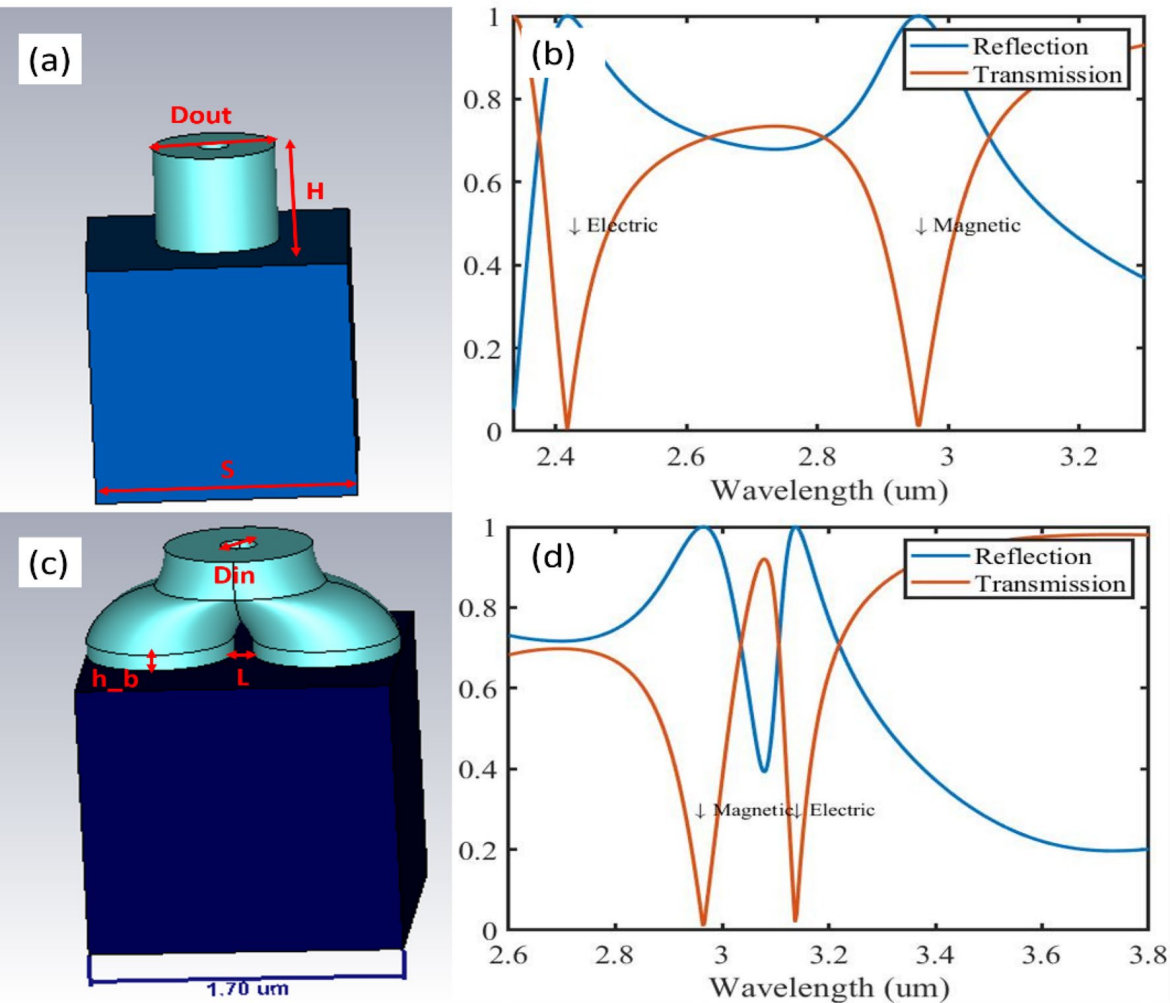
(**a**) Conventional cylinder (**b**) cylinder transmission and reflection (**c**) BPC (**d**) BPC transmission and reflection. The words electric and magnetic refer to the type of dipoles present.

**Figure 2:**
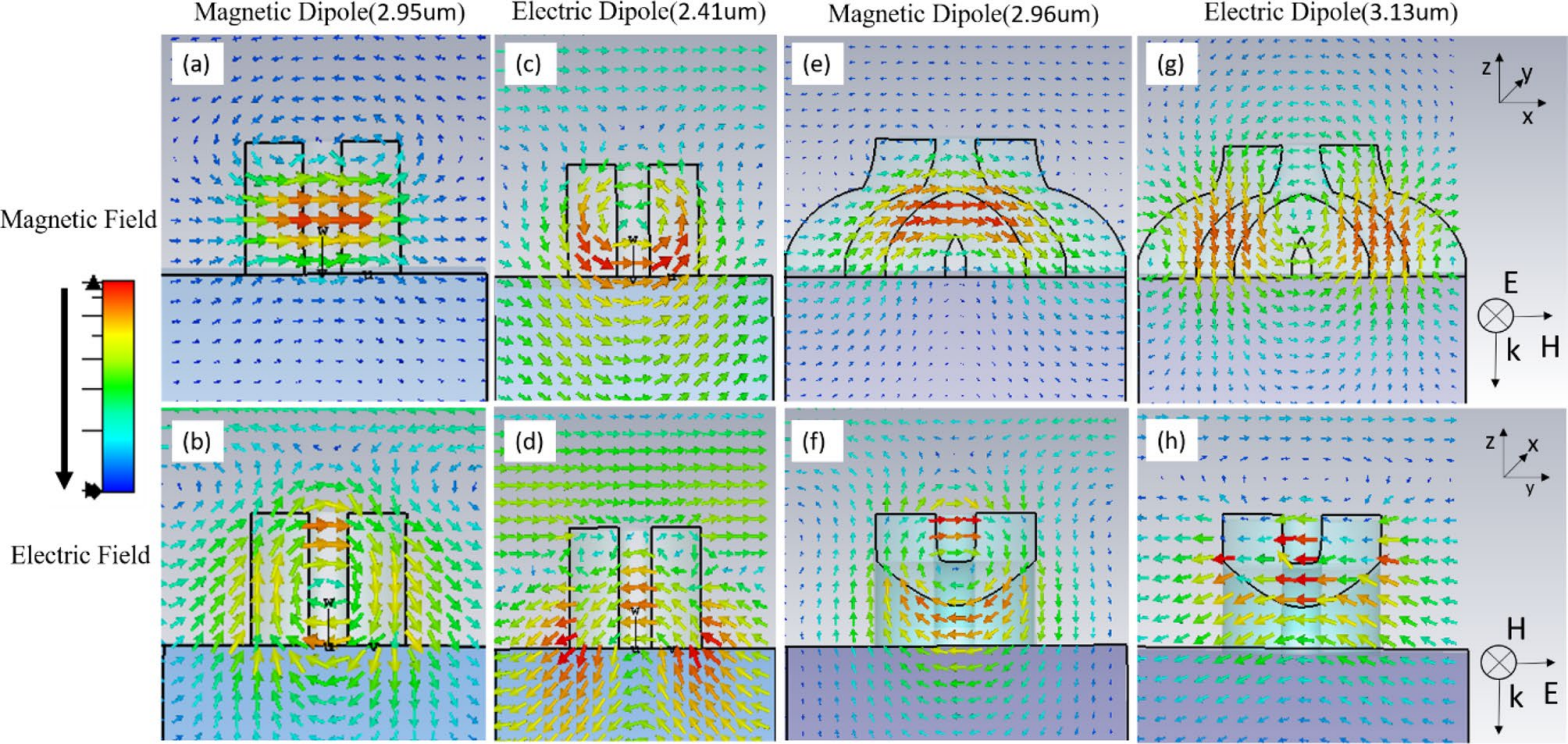
Absolute intensity of magnetic and electric fields represented by arrows, their corresponding dipole modes and wavelengths for conventional cylinder (**a**,**b**,**c**,**d**) and BPC (**e**,**f**,**g**,**h**).

As a first step towards investigating and understanding the electromagnetic behaviour of the proposed BPC unit cell, we compare its performance to a conventional cylinder. Figure 1a,c show the geometry of the cylinder and BPC respectively, with the parameters mentioned above. Figure 1b,d show the magnitude of the transmission and reflection coefficients for the cylinder and BPC respectively. We observe two sharp resonances for each structure, closer together in BPC than in the cylinder. For the cylinder unit cell, by checking the electric and magnetic field intensities maps represented by the arrows shown in Fig. 2a–d we observe the two lowest Mie resonances: TE_011_ and TM_011_ corresponding to magnetic and electric dipoles^18,19^ (MD and ED) respectively. For the MD at 2.95 µm, we see a high intensity magnetic field localized at the centre of the cylinder (Fig. 2a) caused by the displacement current resulting from the looping electric field (Fig. 2b), while the ED at 2.41 µm, is caused by the polarizability of the material in response to the excitation field (Fig. 2c,d). The magnetic field in Fig. 2c is distributed along the lower side of the walls with two maxima at each wall and thus the ED mode is termed distributed ED^15^. Figure 2e–h shows the absolute intensity of the magnetic and electric fields causing the resonances in BPC. We observe similar behaviour to that of the conventional cylinder, however the ED occurs at a larger wavelength than the MD, unlike the conventional cylinder, which can be explained by the perturbation introduced to the structure by the addition of an extra leg along with a gap between the two bottom legs. This may provide a road map for tailoring the resonance frequencies^20,16^. It is also observed that the ED mode shifts to higher frequency closer to MD in case of BPC, which could be explained by the presence of two magnetic field maxima along the two legs of BPC (Fig. 2g) resulting in a coupled and stronger ED mode^21^. Contour plots of the fields are also shown in Supplementary Fig. S1.

**Figure 3:**
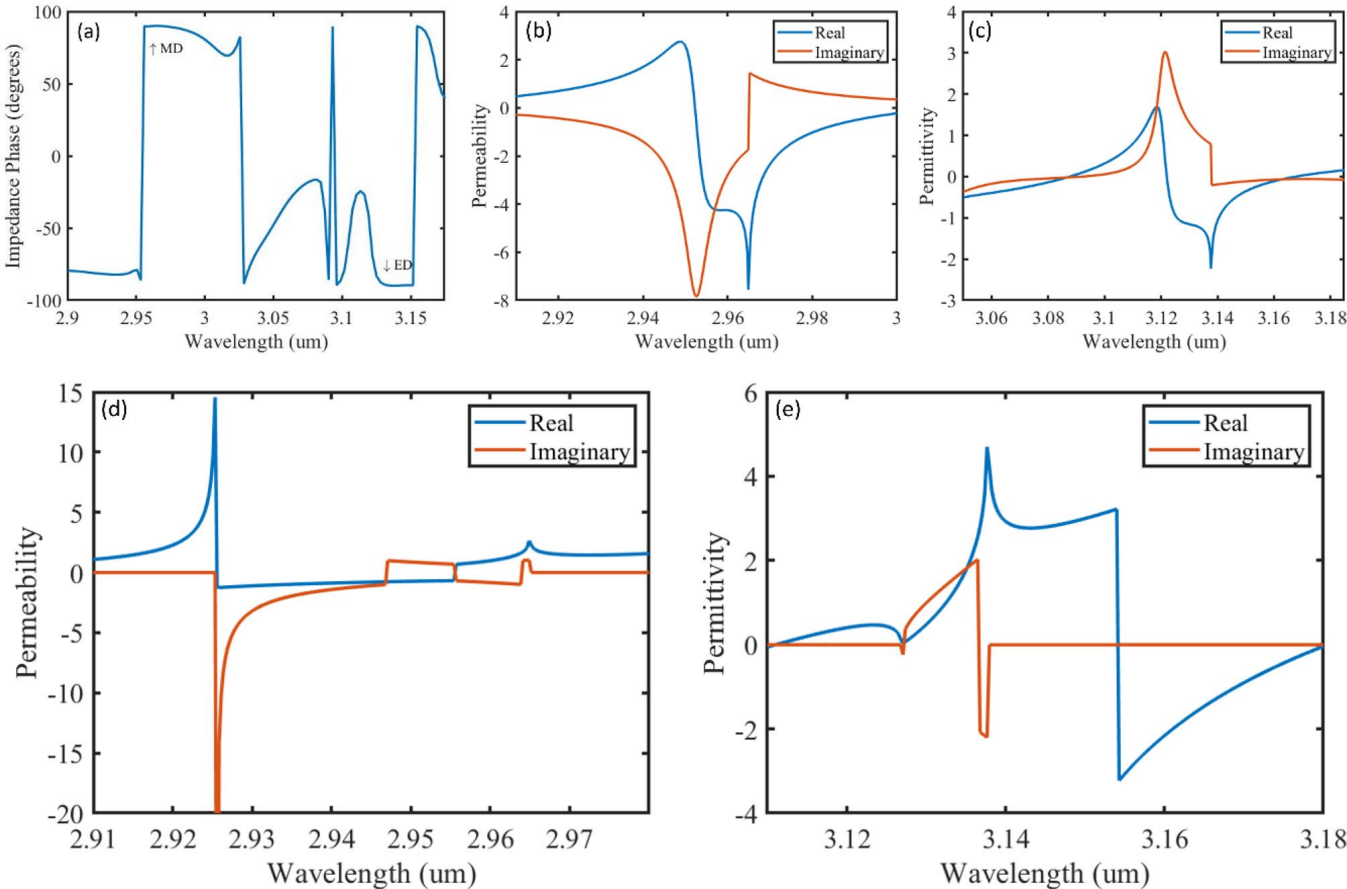
Dispersion parameters for positive propagation: (**a**) impedance phase (**b**) effective permeability (**c**) effective permittivity, and dispersion parameters calculated from averaged s-parameters: (**d**) effective permeability (**e**) effective permittivity.

Next, we discuss the dispersion behaviour of the BPC. For a wave propagating from the positive z direction, the retrieved parameters are shown in Fig. 3 following the procedure described by Smith et al.^22^. We expect the impedance phase of the BPC to be positive at MD, where the permeability (µ) is negative (Fig. 3b), and to be negative at ED where the permittivity (ε) is negative (Fig. 3c)^23^. The impedance becomes purely imaginary (± 90°) at the described wavelengths, meaning that the structure acts as nonideal magnetic and electrical conductors at MD and ED, respectively. However, although |S_11_| =|S_22_|, their real and imaginary parts are different. This is expected because BPCs are asymmetric with respect to the excitation direction. The structure is also considered inhomogeneous given the ratio between the wavelength to the unit cell dimensions, making the parameters in Fig. 3 effectively valid for one-way propagation only. The theory of periodic structures predicts the possibility of replacing the BPC with a homogeneous slab of unique parameters using an averaged S parameter (${S}_{average}=\sqrt{{S}_{11}\times {S}_{22}}$). The effective material parameters are extracted using ${S}_{average}$ and shown in Fig. 3d,e^22,24,25^. The negative permeability is obtained at 2.927 µm and the negative permittivity is obtained at 3.155 µm. This shows a very good agreement with the simulated results which predicted the negative permeability and negative permittivity to be at 2.961 µm and 3.134 µm respectively. It is worth mentioning here that effective medium approximations are most adequate in describing structures of dimensions much smaller than the excitation wavelength^26^, while the thickness of our unit cell is only slightly smaller than the excitation wavelength. In addition, resonances disrupt the dispersion parameters. Moreover, we have previously shown that fabricated multipodal nanotubes (tubes with more than one leg) as well as structures of similar tapered-like geometry possess graded refractive index^27–30^. All these reasons explain the slight discrepancy in the resonance frequencies calculated from the averaged s-parameters, as well as the artefacts present in the imaginary parts of ε and µ.

It is important to discuss the fabrication feasibility at this point. We have previously fabricated bipodal and even multipodal nanotubes from an alloy of Titanium–Niobium–Zirconium via facile electrochemical anodization^27,28^. Mohammadpour et al. as well as Naduvath et al have fabricated multipodal nanotubes of Titanium Dioxide through anodization as well^31,32^. It has been shown that it is possible to etch silicon nanotubes with very specific dimensions using oxygen plasma etching^33^. Moreover, Chen et al. were able to fabricate mutipodal Silicon Nanotubes via Anodic Aluminum Oxide template-assisted Approach^34^. Not to mention that with the use of additive manufacturing and with the presence of rapidly growing technologies like nano-scale 3D printing^35^, the fabrication of BPCs seems very feasible.

### Effect of design parameters on MD and ED

**Figure 4:**
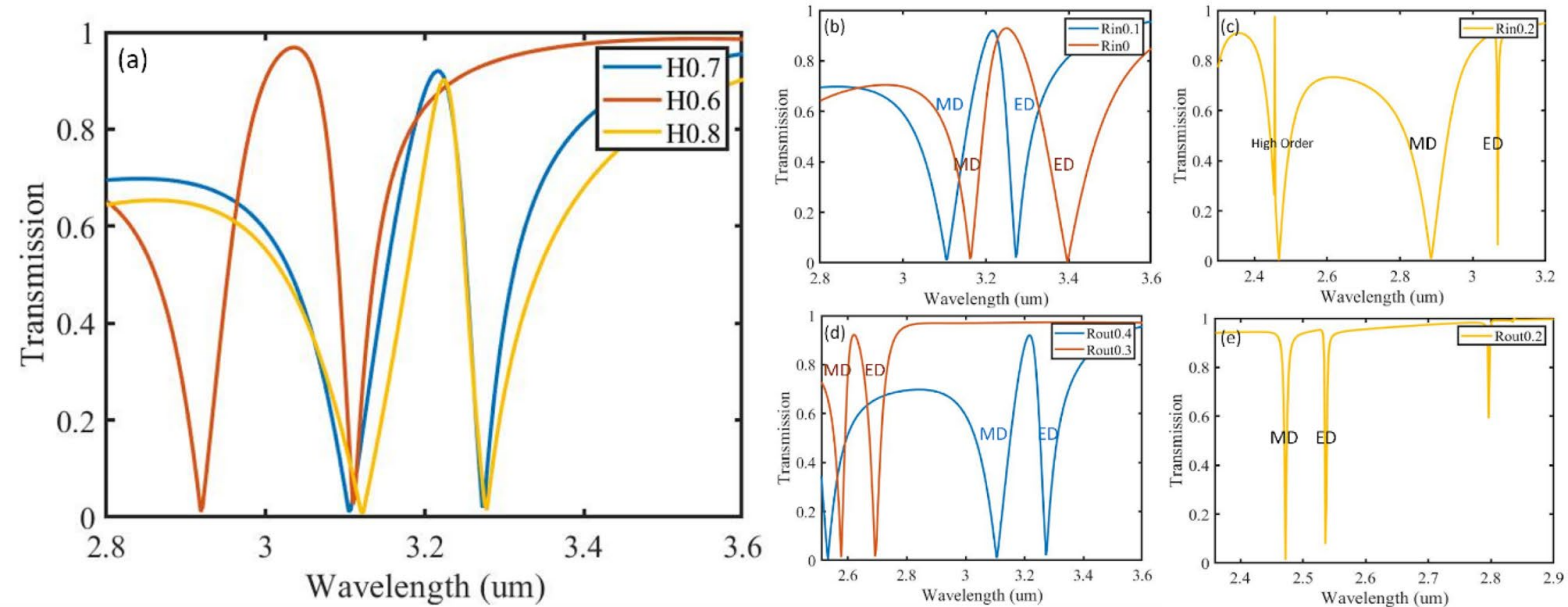
Transmission spectra of BPCs of (**a**) heights 0.6, 0.7, 0.8 µm (**b**,**c**) constant outer radius of 0. µmand inner radii of 0, 0.1, 0.2 µm (**d**,**e**) constant inner radius of 0.1 µm and outer radii of 0.4, 0.3, 0.2 µm.

In this section, we proceed to study the effect of various geometrical parameters on the electromagnetic performance of BPC. Figure 4 summarizes the effect of changing the design parameters: height, outer and inner radii of the BPC, all other parameters are kept the same. In Fig. 4a, a blue shift is observed in the transmission spectra for the BPC of height 0.6 µm (H0.6) compared to that of heights 0.7 µm and 0. 8 µm (H0.7 and H0.8) because a smaller fraction of the current loops can now be present in a smaller height BPC. The three heights were found sufficient to support the build-up of oppositely oriented electric fields, which give rise to a displacement current that can produce MD mode, while ED on the other hand is almost always present because of the uniform magnetic permeability inside and outside of the BPC. Furthermore, it was found that increasing the height decreases the spectral separation between ED and MD modes (Supplementary Table S1).

Figure 4b,c shows the effect of varying the inner radius at constant outer radius of 0.4 µm, where Rin0, Rin0.1 and Rin0.2 correspond to inner radii of 0, 0.1 and 0.2 µm respectively, i.e., wall thickness of 0.4, 0.3, and 0.2 µm. As expected, a blue shift occurs as the wall thickness is decreased owing to the decreased effective refractive index. When the wall thickness is 0.2 µm (Fig. 4c), higher order modes start to appear, and ED mode is very sharp, which could be attributed to the small particle size^1^. Figure 4d,e show the transmission spectra when varying the outer diameter, same blue shift is observed for smaller wall thickness, both resonances are very sharp at wall thickness of 0.1 µm. However, although Rin0.2 and Rout0.3 have the same wall thickness of 0.2 µm, their volumes and surface areas are different, which result in different volumetric filling factors giving different refractive indices, and hence scatter light differently.

### Beam steering structure

**Figure 5:**
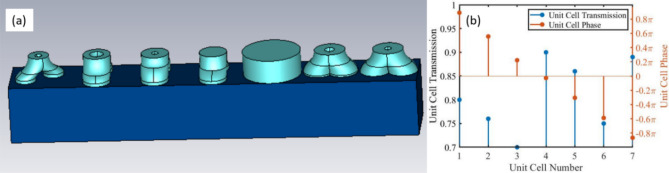
(**a**) The beam steering structure (**b**) Transmission magnitude and phase of each cell.

To form the phase gradient beam steering structure, a parametric study over the BPC parameters was performed in order to find seven unit cells whose transmission magnitudes are 0.7 or higher and whose transmission phases cover the range 0–2π. The height of the BPC is 0.7 µm, chosen to be sufficient for the build-up of opposing fields and thus induce MD and ED around 3 µm as explained above, and the operation wavelength is chosen to be 3.1 µm. The unit cell size is 1.7 µm, only slightly larger than the requirement of the size being subwavelength in the transverse direction (λ/2 or less)^2,36,37^. The parameters of each unit cell are summarized in Table 1 and the unit cells are shown in Fig. 5a. Figure 5b shows the magnitude of the transmission coefficient for each of the unit cells and the associated phase.

**Table 1:** Parameters of the cells used to form the beam steering structure; all dimensions are in µm.

Cell number	D_out_	D_in_	L	RC	h_b
1	0.3	0.1	0.45	0.5	0.1
2	0.4	0.2	0.45	0.5	0.1
3	0.4	0.1	0.45	0.5	0.1
4	0.4	0	0.45	0.5	0.1
5	0.85	0	0	0	0
6	0.45	0.15	0.35	0.5	0.05
7	0.45	0.1	0.4	0.5	0

**Figure 6:**
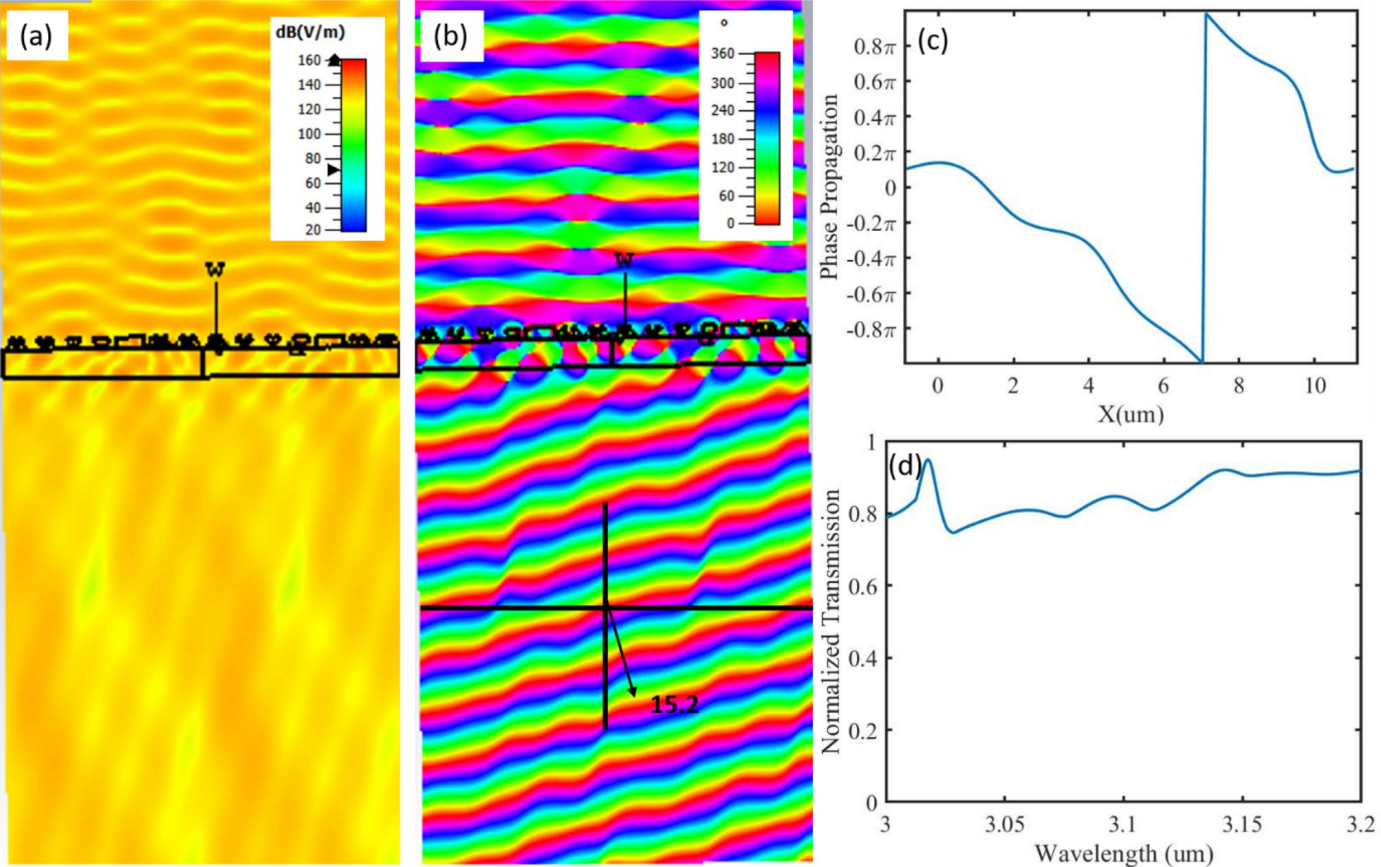
(**a**) Log scale of the electric field intensity (**b**,**c**) electric field phase propagation along the x-direction (**d**) normalized transmission of the metasurface.

The phase gradient results in modifying Snell’s law, which we can use to find the angle of transmission at normal incidence by use of Eq. (1), to be approximately 15 degrees^38,38^.

1 $$\theta _{t} = ~\left( {\frac{{n_{1} sin~\theta _{1} ~ + ~\frac{{\lambda _{o} }}{\Gamma }}}{{n_{t} }}} \right)~,$$

where $\theta _{t}$ is the transmission angle along the transverse direction, $n_{1}$ and $n_{t}$ are the air refractive index (1),$~\theta _{1}$ is the angle of incidence, $\lambda _{o}$ is the operation wavelength, $\Gamma$ is the periodicity (11.9). Figure 6 shows the electric field log scale intensity (Fig. 6a) as well as the phase propagation (Fig. 6c) and transmission (Fig. 6d). We can see that high Transmission efficiency is obtained (0.84), and with high accuracy. The simulation shows a steering angle of 15.2° compared to the theoretically predicted value of 15°. It is worth mentioning that the design has some flexibility, using the same unit cells, we can achieve beam steering at wavelengths 3 µm and 3.14 µm as well, with high transmission and steering angle of approximately 11.8° and 12.4°, respectively (Supplementary Fig. S2).

## Conclusion

We presented a novel structure known as bipodal cylinders and studied its electromagnetic behaviour. The dispersion behaviour of the BPC was investigated. We observed a spectral cross over in its resonance modes compared to conventional cylinders. We numerically showed the possibility to form a beam steering metasurface with high transmission based on BPC unit cells.

## Supplementary Information

Supplementary Information.
