# Supplementary material for: Novel silicon bipodal cylinders with controlled resonances and their use as beam steering metasurfaces
Source: Sci Rep. 2021 Jul 1;11:13635. doi: 10.1038/s41598-021-93041-x (PMC8249426; doi:10.1038/s41598-021-93041-x)
Supplement: Supplementary file 1 — Supplementary Information. [file 41598_2021_93041_MOESM1_ESM.docx]

**Supplementary Information**

**Novel Silicon Bipodal Cylinders with Controlled Resonances and Their Use as Beam Steering Metasurfaces**

**Samar M. Fawzy**^1,3^**, Ahmed M. Mahmoud**^1^**,** **Yehea I. Ismail**^1,2^**, Nageh K. Allam**^3, *^

^1^ Department of Electronics & Communications Engineering, School of Sciences & Engineering, The American University in Cairo, Cairo, 11835, Egypt

^2^Center of Nanoelectronics and Devices (CND), Zewail City of Science Technology and Innovation, Cairo, 12578, Egypt

^3^Energy Materials Laboratory, School of Sciences & Engineering, The American University in Cairo, Cairo, 11835, Egypt.

*Corresponding Author email: nageh.allam@aucegypt.edu


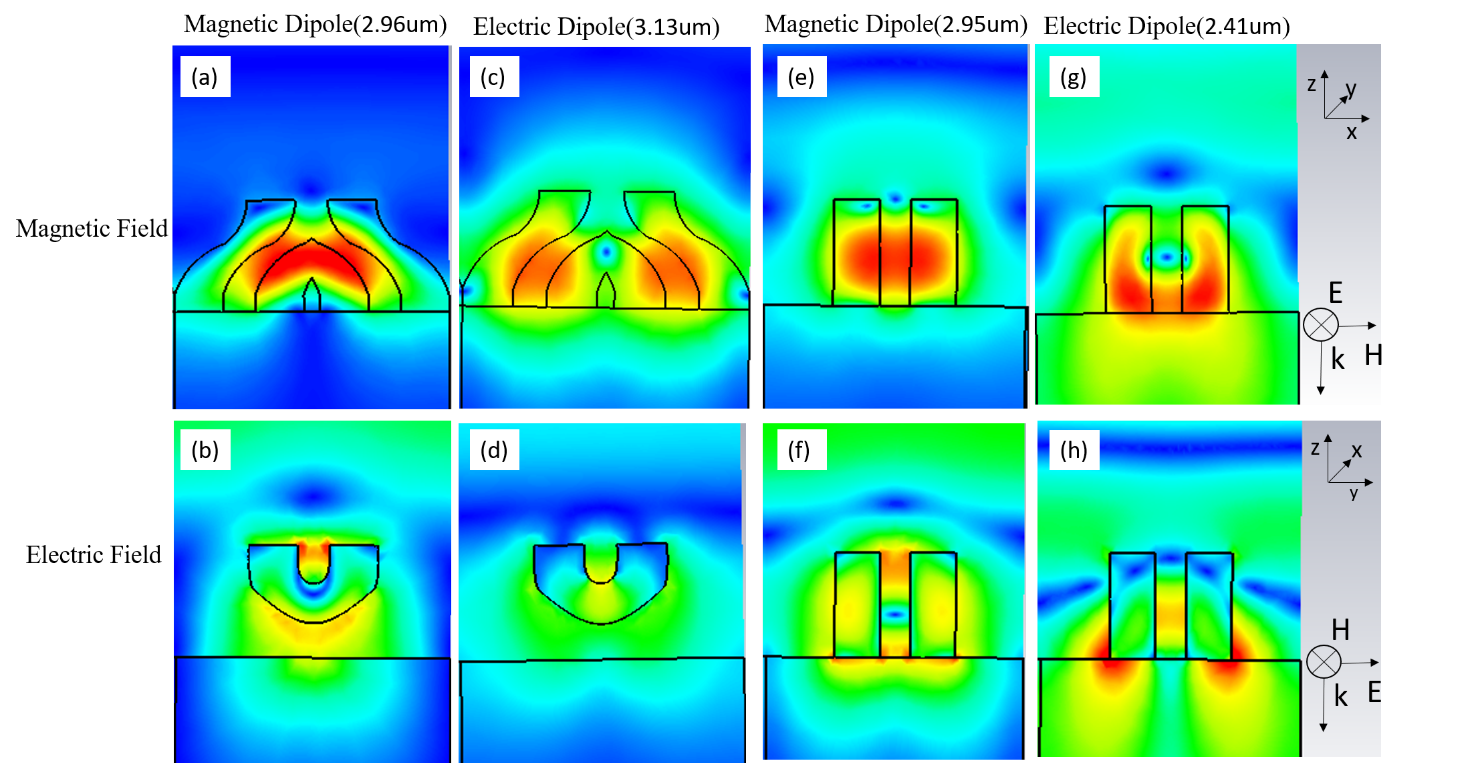


Figure S1 Contour plots of absolute intensity of the magnetic and electric fields, their corresponding dipole modes and wavelengths for conventional cylinder (a,b,c,d) and BPC (e,f,g,h)

Table S1 Separation between MD and ED for BPCs of different heights, inner and outer radii, unit cells in red have the same wall thickness but different inner and outer radii, unit cells in blue are different names for the same cell

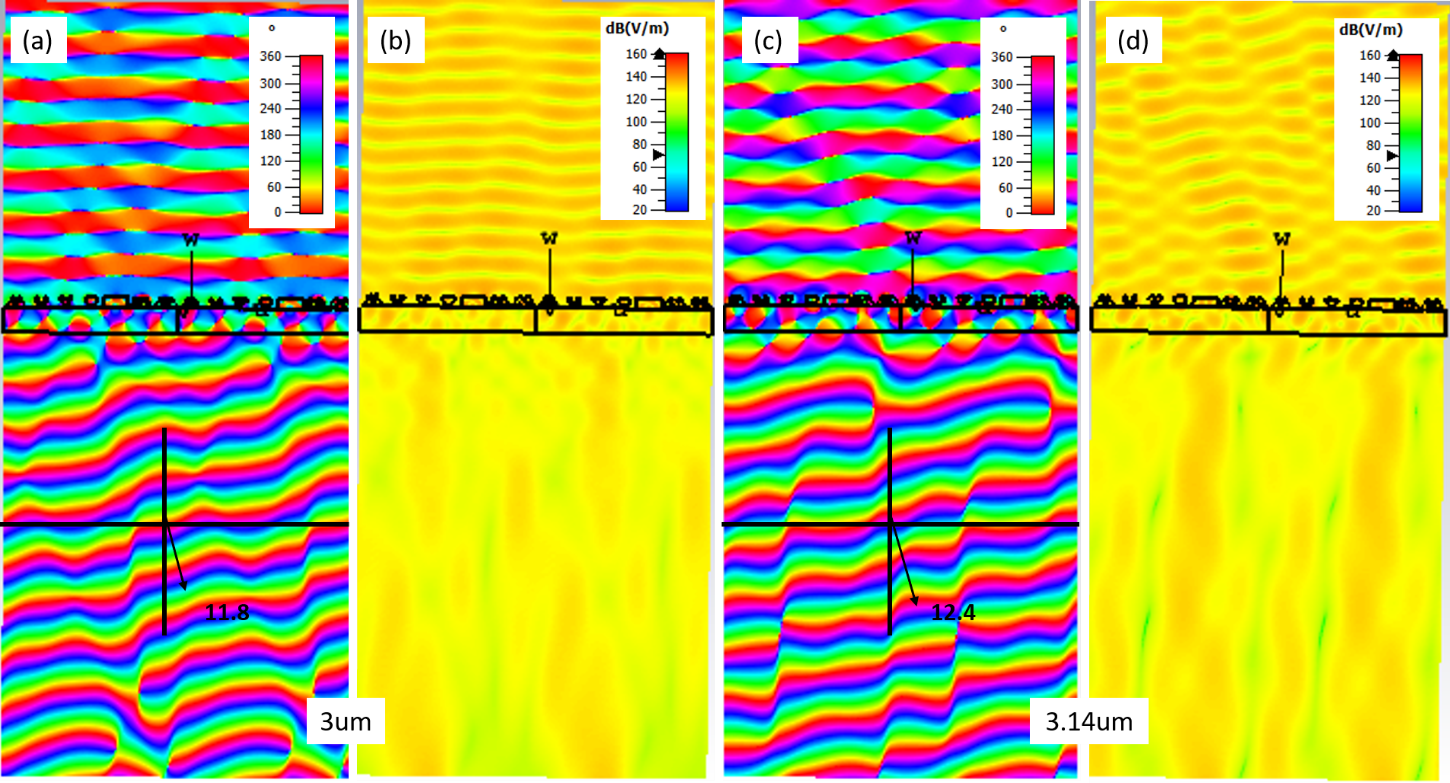


Figure S2 Beam steering at wavelengths 3um and 3.14um using the same structure indicating flexibility of the design
